# RNA-Binding Proteins in the Regulation of Adipogenesis and Adipose Function

**DOI:** 10.3390/cells11152357

**Published:** 2022-07-31

**Authors:** Pengpeng Zhang, Wenyan Wu, Chaofeng Ma, Chunyu Du, Yueru Huang, Haixia Xu, Cencen Li, Xiaofang Cheng, Ruijie Hao, Yongjie Xu

**Affiliations:** 1 Department of Biotechnology, College of Life Sciences, Xinyang Normal University, Xinyang 464000, China; ppzhang@xynu.edu.cn (P.Z.); wwy@xynu.edu.cn (W.W.); 2020210907@xynu.edu.cn (C.D.); huangyr@xynu.edu.cn (Y.H.); hxxu@xynu.edu.cn (H.X.); licencen@xynu.edu.cn (C.L.); chengxiaofang@xynu.edu.cn (X.C.); 2 Institute for Conservation and Utilization of Agro-Bioresources in Dabie Mountain, Xinyang Normal University, Xinyang 464000, China; 3 Xinyang Center for Animal Disease Control and Prevention, Xinyang 464000, China; zhanggavin@webmail.hzau.edu.cn

**Keywords:** RNA-binding protein, post-transcriptional regulation, adipose

## Abstract

The obesity epidemic represents a critical public health issue worldwide, as it is a vital risk factor for many diseases, including type 2 diabetes (T2D) and cardiovascular disease. Obesity is a complex disease involving excessive fat accumulation. Proper adipose tissue accumulation and function are highly transcriptional and regulated by many genes. Recent studies have discovered that post-transcriptional regulation, mainly mediated by RNA-binding proteins (RBPs), also plays a crucial role. In the lifetime of RNA, it is bound by various RBPs that determine every step of RNA metabolism, from RNA processing to alternative splicing, nucleus export, rate of translation, and finally decay. In humans, it is predicted that RBPs account for more than 10% of proteins based on the presence of RNA-binding domains. However, only very few RBPs have been studied in adipose tissue. The primary aim of this paper is to provide an overview of RBPs in adipogenesis and adipose function. Specifically, the following best-characterized RBPs will be discussed, including HuR, PSPC1, Sam68, RBM4, Ybx1, Ybx2, IGF2BP2, and KSRP. Characterization of these proteins will increase our understanding of the regulatory mechanisms of RBPs in adipogenesis and provide clues for the etiology and pathology of adipose-tissue-related diseases.

## 1. Introduction

Adipose tissue has been considered a passive tissue for energy storage. Nowadays, it is emerging as an active participant in regulating whole-body metabolic processes, including lipogenesis, fatty acid oxidation, and lipolysis [1]. In addition, adipose tissue not only responds to hormones from endocrine systems but also secretes factors such as leptin, adiponectin, and resistin, which have been implicated to play key roles in energy homeostasis [2]. Thereby, adipose tissue is recognized as an important organ with both metabolic and endocrine functions.

White adipose tissue (WAT) and brown adipose tissue (BAT) are two main types of adipose tissue. WAT is mainly distributed in the subcutaneous and abdominal organs [3]. WAT is the main location for energy storage in the form of large lipid droplets, which account for more than 90% of cell volume [4]. BAT is mainly distributed in the neck and scapula of newborn animals. Brown adipocytes are multi-chambered with many small lipid droplets scattered with abundant mitochondria in the cytoplasm [5]. In contrast to the energy storage function of WAT, BAT has a very strong fatty acid oxidative ability and dissipates energy as heat [6]. Recently, a new type of adipocyte, beige adipocyte, was discovered in a certain area of WAT [7]. At the basal state, beige adipocytes are morphologically indistinguishable from white adipocytes and have low thermogenesis activity. However, once they are subjected to certain stimuli, such as cold exposure or β-adrenergic stimulation, the beige adipocytes will undergo browning progress [8]. The beige adipocytes start to express BAT-specific genes and transform into the phenotype closer to brown adipocytes, producing more mitochondria and heat [9]. Indeed, increasing BAT mass and activating the browning process have been considered possible strategies to combat obesity and antidiabetic effects [10].

## 2. Transcriptional Control of Adipogenesis

**Figure 1:**
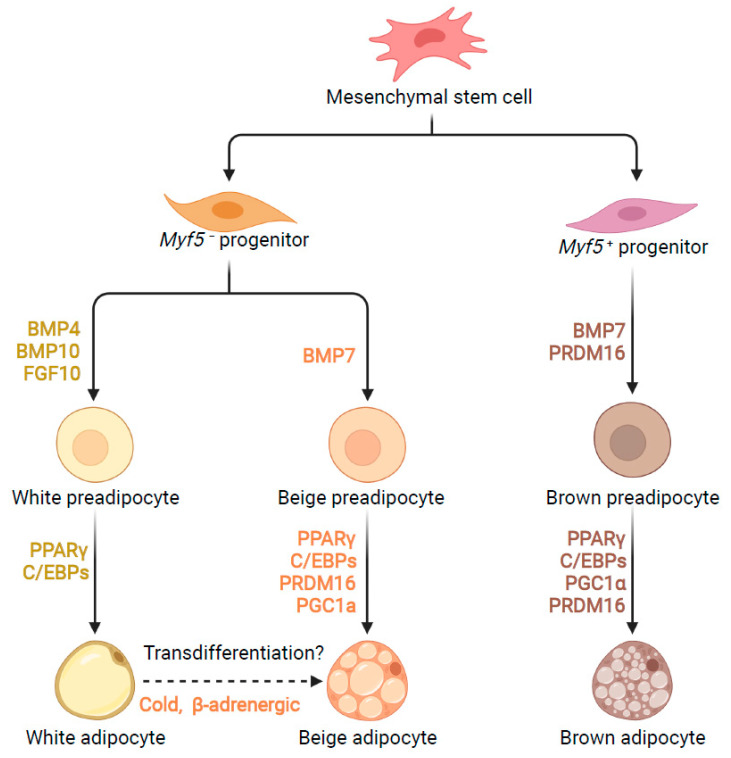
Transcriptional control of adipogenesis. All types of adipocytes are derived from mesoderm mesenchymal stem cells, which contain Myf5^−^ and Myf5^+^ cell lineage. FGF10, BMP4, and BMP10 stimulate Myf5^−^ cell commitment to white pre-adipocyte, while BMP7 triggers Myf5^−^ cell commitment to beige adipocyte. BMP7 and PRDM16 induce Myf5^+^ cell commitment to brown pre-adipocyte. PPARγ and C/EBPs play key roles in all types of adipocyte differentiation. Brown and beige adipogenesis require the expression of additional proteins, including Prdm16 and Pgc1α. In addition, beige adipocytes may also come from transdifferentiation of white adipocytes under cold exposure or β-adrenergic stimulation.

The growth of adipose tissue involves the formation of new adipocytes from precursor cells, termed adipogenesis. Once adipogenesis is limited, lipid continuously accumulates and finally results in adipocytes rupturing and causing inflammation and insulin resistance [11]. Thus, adipogenesis is an important process related to health. Though all types of adipocytes are derived from mesoderm mesenchymal stem cells (MSCs), they belong to a distinct cell lineage [12]. MSCs contain both Myf5^−^ and Myf5^+^ cell lineage. White and beige adipocytes mainly come from Myf5^−^ precursor cells, while BAT adipocytes come from Myf5^+^ precursor cells. A series of excellent reviews has provided details on the transcriptional regulation of adipogenesis [13,14,15]. Briefly, the process of adipogenesis is comprised of two phases: the determination phase and the terminal differentiation phase [16]. Although white, beige, and brown adipocytes are originally and functionally distinct, the adipogenesis for all of them requires general adipogenic machinery, including CCAT/enhancer-binding protein (C/EBP) family members and peroxisome proliferator-activated receptor-γ (PPARγ). In the early determination phase, the expressions of C/EBPβ and C/EBPδ are induced [17,18]. The mesenchymal stem cells commit to becoming pre-adipocytes. Though they cannot be distinguished morphologically, pre-adipocytes have lost the ability to differentiate into other cell types [19]. In the terminal differentiation phase, C/EBPβ and C/EBPδ activate the expression of PPARγ and C/EBP-α [20]. The pre-adipocytes further differentiate and acquire the characteristics of the mature adipocytes. PPARγ and C/EBP-α have been shown to play central roles in regulating adipogenesis, as force expression of each gene can induce the differentiation of many fibroblastic cell lines into adipocytes [21]. In addition, brown and beige adipogenesis also require specific transcription factors, including Prdm16 and Pgc1α (Figure 1) [15].

## 3. Post-Transcriptional Control of Adipogenesis

**Figure 2:**
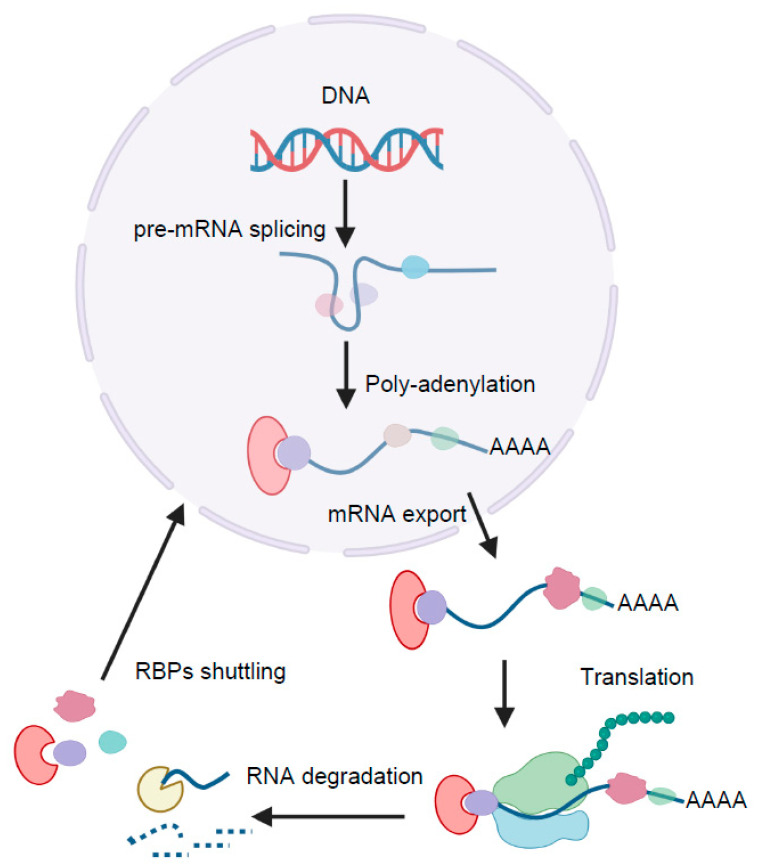
Post-transcriptional regulation of RNA by RNA-binding proteins (RBPs). RNA binds with various dynamically changed RBPs (color shapes) across its lifetime. RBPs are involved in alternative splicing, polyadenylation, RNA transport, RNA stability, the rate of translation, and finally the levels of protein produced.

Although transcriptional regulation is indispensable in adipogenesis, post-transcriptional regulation has been implicated in increasing importance [22,23]. RNA-binding proteins (RBPs) are recognized to play a central role in gene post-transcriptional regulation, both in various physiological or pathological processes [24]. Usually, a typical RBP binds to hundreds of RNA transcripts based on a specific sequence and/or RNA structure. In humans, 2000 proteins are predicted to be RBPs based on the presence of RNA-binding domains [25]. Though the functions and RNA targets for most RBPs are unknown, the characterized RBPs participate in every step of RNA life. Once the pre-mRNAs are transcribed, they are associated with dynamically changed RBPs. RBPs are involved in alternative RNA splicing, polyadenylation, RNA transport, RNA stability, the rate of translation, and finally the levels of protein produced. In addition, RNAs are bound by a variety of RBPs, which coordinate with each other to post-transcriptionally control the processes of RNA (Figure 2) [26,27].

## 4. Regulation of Adipogenesis and Adipose Function by RBPs

RBPs play key roles in fundamental cellular activities, including proliferation, differentiation, and apoptosis. Dysregulation of RBPs leads to a variety of diseases, such as cancer [28], myopathies [29,30], and neurological [31] diseases. Although a large number of RBPs have been identified to mediate RNA metabolism, only a few of them have been studied in adipose tissue. Recently, several RBPs have been proved to play essential roles in adipogenesis. In addition, some RBPs are mutated or dysregulated in obesity and diabetes [32]. Thereby, a summary of these RBPs will facilitate the related study and can provide new strategies for weight management and obesity-related diseases. In the current review, we will describe the role of HuR, PSPC1, Sam68, RBM4, Ybx1, Ybx2, IGF2BP2, and KSRP in adipogenesis and discuss how they regulate RNA activities.

### 4.1. HuR

HuR is a member of the ElAV-1 family. Unlike other members of ElAV-1 family (HuB, HuC, and HuD), which are primarily expressed in neuronal cells, HuR is ubiquitously expressed in various tissues [33]. HuR can bind to AU-rich or U-rich elements of target RNAs via its three evolutionarily conserved RNA recognition motifs (RRMs) (Table 1) [34]. HuR contains a nucleocytoplasmic shuttling sequence (HNS) between the second and third RRM domains. Post-transcriptional modification of the HNS controls the translocation of HuR between the nucleus and the cytoplasm [35]. The function of HuR is dependent on the dynamic subcellular localization. Once HuR shuttles from the nucleus to the cytoplasm, HuR increases the stability and translation of target RNAs [36]. In addition, HuR has been demonstrated to regulate other stages of the RNA process, including alternative splicing, RNA translocation, and polyadenylation [33].

HrR is proven to function in regulating cellular proliferation and differentiation. At first, several of cycle-regulatory genes, including p21 and cyclins A and B, were identified as the HuR targets [37,38]. HuR stabilizes the RNAs and promotes their protein levels, thereby regulating cell proliferation. Further, HuR has been discovered to modulate differentiation. When HuR shuttles from the nucleus to the cytoplasm, it stabilizes and increases RNA levels of MyoD and myogenin, thus inducing myogenesis [39]. Consistent with this, knock down of HuR inhibits myoblast differentiation by down-regulating MyoD and myogenin expression [40].

**Figure 3:**
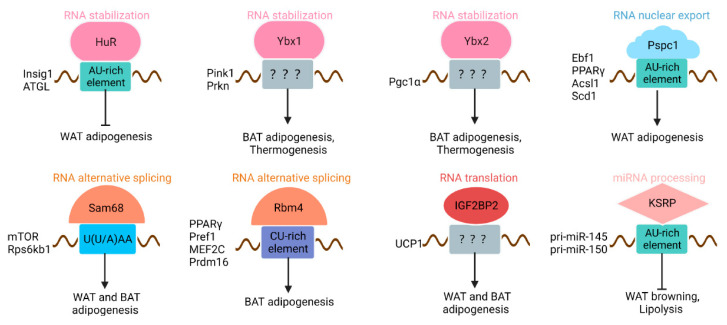
Functions of the selected RNA-binding proteins (RBPs) in adipose tissue. The RNA targets and the cis elements bound by RBPs are shown. The shape of RBP indicates a specific effect on RNA targets.

Recently, HuR has been shown to post-transcriptionally regulate multiple genes that play vital roles in adipogenesis. In obesity and type 2 diabetes mice, the expression level of HuR decreases in both WAT and BAT [41]. Accordingly, the expression of HuR also drops during the adipocyte differentiation process in vitro. Li and colleagues have identified that HuR negatively regulates adipogenesis [42]. Knockdown of HuR promotes primary white and brown adipocyte differentiation, while overexpression of HuR inhibits differentiation, suggesting the inhibiting role of HuR in adipogenesis. Consistent with the function of HuR in vitro, HuR knockout mice show an enlarged fat mass. By performing RIP-seq, insulin-induced gene 1 (Insig1), which negatively regulates adipogenesis [43], is identified as one of the top binding targets of HuR (Figure 3). HuR can bind to Insig1 and increase the stability of the RNA, resulting in impaired adipogenesis [42]. When mice are fed a high-fat diet, the HuR knockout mice show an obesity phenotype and suppressed lipolysis. Mechanically, HuR directly interacts with the ATGL transcript and promotes its stability and protein level. Together, these results establish that HuR regulates adipogenesis and lipid accumulation by modulating RNA stability. Future investigations may identify more AU-rich or U-rich RNAs that are post-transcriptionally regulated by HuR and involved in adipogenesis. In other tissue, HuR also functions in alternative splicing [44], RNA translocation [45], and polyadenylation [46]. It remains to be explored whether HuR regulates adipogenesis in these ways.

### 4.2. PSPC1

PSPC1 belongs to the Drosophila behavior/human splicing (DBHS) family. DBHS proteins have two highly conserved RRMs at N-terminal. Members of DBHS have been suggested to bind a variety of single-stranded RNA, conserved 5′ splice sites, and U5 snRNA stem-loops (Table 1) [47]. DBHS proteins are ubiquitous in animals. Lower vertebrates usually encode one or two DBHS members, whereas humans encode three DBHS proteins, including SFPQ, PSPC1, and NONO. Crystal structure analysis suggests that DBHS proteins rarely function alone. The three human DBHS proteins form homodimers or heterodimers among each other [48]. DBHS proteins have multiple roles in RNA metabolism, such as transcription, splicing, and translocation [49].

The DBHS proteins transcriptionally and post-transcriptionally regulate genes participating in multiple processes, such as tumorigenesis [50] and neurological development [51,52]. Compared to other members of DBHS proteins, the role of PSPC1 is less known. Recently, PSPC1 has been proved to play a vital role in adipogenesis [53]. During pre-adipocyte differentiation, the expression of PSPC1 is continually induced by PPARγ, which directly binds to the promoter of PSPC1 and activates transcription. Gain-of-function studies showed that PSPC1 in pre-adipocytes promotes adipogenesis, while knockdown of PSPC1 impairs adipogenesis and diminishes lipid accumulation [53]. Consistent with the function in vitro, mice deleted with PSPC1 show less fat mass and lipid storage. Further, mutation of the PSPC1 RRMs motif renders the adipogenic capacity of PSPC1, suggesting the function of PSPC1 in adipogenesis depends on RNA binding. Additionally, genome-wide RNA targets of PSPC1 were identified by iCLIP-seq. It was found that the PSPC1 binding sites are AU-rich. Several PSPC1 target transcripts play key roles in adipogenesis, such as Ebf1, PPARγ, Acsl1, and Scd1 (Figure 3). In addition, PSPC1 interacts with the RNA export factor DDX3X, promoting the nuclear export of the target RNAs, including SCD-1 and PPARγ [53]. These results demonstrate the vital roles of PSPC1 in RNA transport and adipose development. According to previous studies, the DBHS protein family usually forms homo- and heterodimers [48]. Thus, it would be interesting to explore whether PSPC1 acts alone or interacts with other DBHS proteins to post-transcriptionally regulate adipogenesis.

### 4.3. Sam68

Sam68 was initially found to be a target of the tyrosine kinase c-SRC [54]. It belongs to the STAR family, which functions in RNA processing. Sam68 contains a KH domain and binds to U(U/A)AA motif (Table 1) [55]. In addition, Sam68 has six proline-rich elements mediating protein–protein interaction and a tyrosine-rich C-terminal, which could be phosphorylated by tyrosine kinases [56]. Furthermore, it was discovered that Sam68 protein could also be methylated, acetylated, and even SUMOylated. Those post-translational modifications affect Sam68 subcellular localization, binding with signaling proteins and target RNAs [57]. Thereby, Sam68 is recognized as a signal transduction and activation of RNA (STAR) protein [56]. Sam68 is implicated in the regulation of transcription, alternative splicing, RNA transport, and mRNA translation [58]. Interestingly, Sam68 is also found to interact with DROSHA and DICER, which are key enzymes in the processing of miRNA, thereby playing a role in the processing of miRNA [59].

Sam68 participates in a variety of cellular processes, such as neurogenesis, cell cycle, and apoptosis [60,61]. Examination of the tissue expression profile revealed that Sam68 is widely expressed. Knockout of Sam68 in mice leads to high lethality, and the viable mice were protected from aging bone mass loss [62]. In addition, ablation of Sam68 impaired spermatogenesis. Sam68 interacts with splicing regulators and thereby results in abnormal alternative splicing [63].

In pre-adipocytes, knockdown of Sam68 leads to adipogenesis deficiency. Consistent with this, the Sam68 knockout mice show a decreased commitment of adipocyte progenitors and less accumulation of adipose tissue, including both WAT and BAT. In addition, the knockout mice are protected from high-fat diet-induced obesity. By performing genome-wide exon expression analysis, a significant number of alternative splicing events are identified. Especially, a shortened mTOR splicing form is generated, and the expression of the functional mTOR protein is inhibited, thereby disrupting mTOR signaling and resulting in adipogenesis defects [64] (Figure 3). Further, sam68 is also implicated in the alternative splicing of Rps6kb1. Knockout of Sam68 leads to the generation of a new transcript isoform Rps6kb1-002, which suppresses adipogenesis and lipid accumulation. In wild-type adipocytes, Sam68 suppresses the generation of Rps6kb1-002 by counteracting with SRSF1. Once Sam68 knockout takes place, SRSF1 inhibits adipogenesis by promoting the generation of Rps6kb1-002 [65]. In addition, Sam68 has been described interacting with lncRNA stability [66]. In pre-adipocytes, Sam68 regulates the stability of several lncRNAs that have essential functions in adipocyte differentiation, such as mir155hg, SR-lncRNA-1, and SR-lncRNA-2. Interestingly, mir155hg is the primary sequence of miR-155, which is an inhibitor of adipogenesis [67]. Together, these studies suggest that Sam68 is a key regulator of adipogenesis. Given the idea that miRNA plays critical roles in adipogenesis and lipid metabolism, together with the involvement of Sam68 in miRNA processing [59], it would be interesting to investigate the function of Sam68 in modulating miR-155 processing in adipogenesis.

### 4.4. RBM4

RBM4 was originally found to function in the circadian rhythm of Drosophila [68]. RBM4 is highly conserved across evolution. The human RBM4 gene shares 95% similarity with that of mice [69]. In mammals, there are two isoforms, RBM4a and RBM4b. The two isoforms are very similar in sequence and are predicted to be generated through gene duplication [70]. Since only RBM4a has been investigated, the current review refers to this isoform. RBM4 has been identified as an RBP that contains two N-terminal RRMs and a zinc finger [71]. The C-terminal is essential for the interaction with other proteins and sublocations [69,70]. The RRMs of RBM4 prefer binding to CU-rich sequences and regulating target RNA alternative splicing [72]. The function of the zinc finger in animals is not determined yet, but research in Drosophila implicated that it may be involved in modulating target RNA translation (Table 1) [73].

RBM4 is expressed ubiquitously [74]. In adipose tissue, the expression of RBM4 increases during brown and white adipocyte differentiation. Knockout of RBM4 leads to impaired brown adipocyte differentiation. Mechanically, RBM4 is involved in modulating alternative splicing of multiple transcripts, such as PPARγ and Pref1, which are key regulators of adipogenesis [75] (Figure 3). PPARγ comprises two splicing variants, PPARγ1 and PPARγ2. PPARγ2, but not PPARγ1, is reported to induce adipogenesis [76]. Overexpression of RBM4a leads to the preference for PPARγ2 expression and promotes brown adipogenesis. Pref-1 is a transmembrane protein that consists of four splice isoforms, including Pref-1A, Pref-1B, Pref-1C, and Pref-1D. Pref-1A and Pref-1B inhibit brown adipogenesis by suppressing C/EBP β/δ expression at the early stage of pre-adipocyte differentiation [77,78]. RBM4a promotes adipogenesis by down-regulating Pref-1A and Pref-1B isoforms and enhancing alternative splicing of Pref-1C and Pref-1D transcripts [75].

Myocyte Enhancer Factor 2C (MEF2C) was originally known as an indispensable protein in myogenesis [79]. Recently, MEF2C was also implicated in regulating adipogenesis [80]. Exon 10 of MEF2C is a cassette exon that encodes the g region of MEF2C. During the development of BAT, RBM4 binds to the CU-rich element and promotes the skipping of exon 10, which enhances the production of MEF2Cg-. MEF2Cg- activates the transcription of PRDM16, BMP7, and C/EBPβ, thus inducing brown adipocyte differentiation.

PRDM16 is a transcription factor that is indispensable for maintaining BAT identity and function [81]. Four alternative splicing transcripts are generated by PRDM16. During the BAT development, RBM4 mediates the expression of the PRDM16 containing exon16, which exerts a more substantial effect on promoting the BAT-related gene expression.

Taken together, these results suggest that RBM4 has a broad spectrum of RNA targets in BAT. In future investigations, it might be interesting to explore the complete RBM4 targets by performing a whole-genome approach.

### 4.5. Y-Box Binding Proteins

Y-box binding proteins (YBXs), including YBX1, YBX2, and YBX3, were originally named for their ability to bind Y-box motif DNA. Y-box binding proteins contain a highly conserved cold-shock domain (CSD), which could respond to cold stress. The CSD is flanked by the N-terminal alanine/proline-rich (A/P) domain and the C-terminal domain [82]. Later, YBXs were identified as RNA-binding proteins. The CSDs and C-terminal domain were implicated in mediating RNA binding (Table 1) [83,84,85]. YBXs bind to a significant number of RNAs and regulate RNA stability and translation [86,87].

YBXs play roles in a variety of processes, such as proliferation, differentiation, and stress response. Recent studies suggested that Y-box binding proteins are also involved in adipogenesis [88,89,90]. Ybx1 is highly enriched in BAT and could be induced upon cold exposure and β-adrenergic agonists treatment. The expression of YBX1 increases during brown adipocyte differentiation. Knockdown of YBX1 leads to impaired brown adipocyte differentiation and decreases the expression of thermogenic genes. Though the mitochondrial number is not affected by YBX1 knockdown, degradation of defective mitochondria is inhibited. It has been discovered that YBX1 directly binds to Pink1 and Prkn mRNAs, two of the critical proteins that positively regulate mitophagy and increase their stability [89] (Figure 3). Thus, loss of Ybx1 results in mitophagy deficiency and impaired BAT thermogenesis.

Like Ybx1, Ybx2 is also enriched in BAT [90]. The expression of Ybx2 increases during WAT browning. Knockdown of Ybx2 robustly reduces lipid accumulation. The Ybx2 knockout mice show reduced BAT mass and impaired thermogenesis. Rip-seq results suggested that Ybx2 could target hundreds of mRNAs that are enriched for mitochondrial functions, including Pgc1α (Figure 3). Upon cold stimulation, Ybx2 could stabilize its target mRNAs [90], thus promoting the BAT thermogenesis program.

These studies suggested that YBX1 and YBX2 are key players in modulating BAT adipogenesis and thermogenesis by affecting RNA stability. Regarding one of the well-characterized functions of YBXs is regulating translation [91,92], and whether these proteins are involved in the translation of target RNA is not yet answered. In addition, the role of Ybx3 in BAT is little known. Considering members of YBXs are highly similar in structure and sequence, the functional interchangeability of these proteins is worth investigating.

### 4.6. IGF2BP2

Insulin-like growth factor (IGF) 2 encodes a critical growth factor regulating growth and development [93]. The expression of IGF2 is finely modulated through transcriptional and post-transcriptional mechanisms [94]. The IGF2 mRNA-binding protein 2 (IGF2BP2) is a highly conserved RBP that consists of two RNA recognition motif (RRM) domains and four hnRNP K homology (KH) domains (Table 1). IGF2BP2 was originally discovered to bind IGF2 mRNA [95], and then many other IGF2BP2 targets were identified, including TRIM54 [96], UCP1, and a subset of genes encoding mitochondrial components [97]. Consistent with the multiple RNA targets, IGF2BP2 is involved in a spectrum of biological processes, such as development, tumorigenesis, and metabolism [98]. Many independent genome-wide association studies (GWAS) have identified more than 100 SNPs in the second intron of the human IMP2 gene. All of the SNPs are highly associated with impaired insulin secretion and type 2 diabetes [99,100,101,102].

Mice globally deleted with IGF2BP2 are lean and highly resistant to high-fat diet-induced obesity, fatty liver, and glucose intolerance [97]. The knockout mice also show better tolerance to cold exposure. Although the mRNA level of UCP1 is comparable with the control littermate, the UCP1 protein level is about two-fold more abundant in knockout mice. Mechanically, IGF2BP2 binds to many transcripts that encode mitochondrial components and inhibit their translation. Specifically, IGF2BP2 binds to untranslated regions of UCP1 and inhibits its translation in BAT [97] (Figure 3). Thus, global deletion of IGF2BP2 promotes UCP1 protein levels, increases energy expenditure, and leads to beneficial metabolic phenotypes. Curiously, tissue-specific deletion of IGF2BP2 in mice results in an unhealthy phenotype. Adult muscle IGF2BP2 knockout mice show reduced skeletal muscle mass and decreased fatty acid oxidation due to a decrease in PPARα mRNA and protein levels [103]. IGF2BP2 knockout in mouse pancreatic β-cells leads to insulin secretion deficiency [104]. Hepatocyte-specific IGF2BP2 knockout results in the diet-induced fatty liver for impairing fatty acid oxidation [105]. How could IGF2BP2 function in so many different tissues? One of the reasons might be that IGF2BP2 binds different RNA targets across tissues. To develop a complete role of IGF2BP2, additional studies will be needed to explore the specific IGF2BP2 targets in various tissues.

### 4.7. KH-Type Splicing Regulatory Protein

The KH-type splicing regulatory protein (KSRP) is identified as a single-strand nucleic acid binding protein [106]. KSRP is located in both the cell nucleus and cytoplasm. The distribution of KSRP is dynamically regulated by cellular stimuli [107]. KSRP binds to AU-rich elements of RNA via its four KH domains [108] and regulates multiple RNA post-transcriptional events, such as alternative splicing [109], RNA decay [108], and translation [110] (Table 1). Interestingly, KSRP also plays a role in miRNA processing [111]. MiRNA is a type of small noncoding RNA that binds to 3′ UTR of mRNA, resulting in mRNA degradation or translation repression [112]. In the nucleus, KHSRP binds to the terminal loop of a group of miRNA precursors and mediates cleaving of pri-miRNA into pre-miRNA. In addition, KSRP increases the transport of pre-miRNA into the cytoplasm. In the cytoplasm, KSRP interacts with Dicer complexes and promotes miRNA maturity [111,113,114].

In mice, the global knockout of KSRP reduces adipose mass by promoting lipolysis and white adipose browning [115,116]. Upon KSRP deletion, the triacylglycerol content is largely decreased, while the adipocyte differentiation is unaffected. Both in fed and fasting conditions, the KSRP knockout mice show elevated lipolysis and fat utilization in WAT. Mechanically, miR-145 is the only miRNA regulated by KSRP in epididymal WAT. KSRP physically interacts with pri-miR-145 and participates in the processing of miR-145 (Figure 3). MiR-145 can directly target and inhibit both Foxo1 and Cgi58, which function in fatty acid lipolysis [117,118]. Thus, knockout of KSRP leads to the down-regulation of mature miR-145 and the elevation of adipose lipolysis [116]. In addition, the same research group identified miR-150 as the only miRNA regulated by KSRP in inguinal WAT. KSRP knockout mice show increased thermogenesis and mitochondrial fatty acid oxidation in inguinal WAT [115]. Further study indicates that KSRP participates in inguinal WAT pri-miR-150 processing. Forced expression of miR-150 directly targets PRDM16 and PGC1α; thereby, knockout of KSRP reduces the expression of miR-150 and attenuates the inhibiting effects of PRDM16 and PGC1α, which promote WAT browning and the metabolism of lipid [115]. Though previous studies showed that KSRP could bind to the terminal loop of pri-miRNAs and affect miRNA processing, the specific mechanism that KSRP affects miR-145 and miR-150 processing in different parts of WAT remains to be investigated.

## 5. Concluding Remarks and Future Perspective

It is without a doubt that post-transcriptional regulation by RBPs is an essential part of adipogenesis. However, compared to other fields, such as tumorigenesis and neurogenesis, the investigation of RBPs in adipogenesis is insufficient. Considering the large number of RBPs, many of them may contribute to adipogenesis regulation despite those mentioned; thereby, there are many opportunities for further study. To date, the regulatory roles of RBPs in adipogenesis have been mainly focused on alternative splicing, RNA transport, RNA decay, and translation. Alternative polyadenylation generates transcripts with different 3′ UTR length. During adipogenesis, a trend towards longer 3′ UTR was discovered [119], which makes them more or less susceptible to the binding of miRNAs or RBPs, thereby affecting adipogenesis [46,120,121]. In addition, over 100 post-transcriptional modifications, such as N6-methyladenosine (m6A), 5-methylcytidine, and inosine, have been found in RNAs [122]. Modifications can regulate mRNA gene expression and stability [123] and were implicated in various cellular processes, including adipogenesis [124,125]. In addition, the critical roles of RNA modifications in diverse processes rely on interactions with RBPs [126]. Interestingly, HuR has been identified to favor binding with the m6A-enriched sites, regulating m6A-containing mRNA stabilization and translation [127,128,129]. However, little study has been performed to examine the role of RBPs in alternative polyadenylation and RNA modification during adipogenesis. Regarding the importance of these post-transcriptional processes in adipogenesis, future work in these fields will largely expand our understanding of RBPs’ regulatory mechanisms in adipogenesis. Furthermore, given the idea that multiple RBPs may share similar binding sites and compete for binding to target RNAs [130,131], determining how multiple RBPs interact with each other and regulate the metabolism of specific RNAs will be crucial. As described above, a unique RBP usually targets thousands of transcripts [132]. However, current work only examined limited targets. Using a global approach to study RBPs has significant implications for the understanding of how RBPs function in adipogenesis and provides clues for combating obesity and diabetes.

## Figures and Tables

**Table 1:** The best-characterized RNA-binding proteins involved in adipogenesis.

RBP	RNA-Binding Domain	Target Cis-Elements	RNA Targets	Effect on RNA Targets
HuR	RRMs	AU-rich element	Insig1, ATGL	Stabilization
PSPC1	RRMs	AU-rich element	Ebf1, PPARγ, Acsl1, and Scd1	Nuclear export
Sam68	KH	U(U/A)AA	mTOR, Rps6kb1	Alternative splicing
RBM4	RRMs, ZF	CU-rich element	PPARγ, Pref1, MEF2C, Prdm16	Alternative splicing
Ybx1	CSD, C-terminal domain	Not determined	Pink1, Prkn	Stabilization
Ybx2	CSD, C-terminal domain	Not determined	Pgc1α	Stabilization
IGF2BP2	RRM, KH	Not determined	UCP1	Inhibit translation
KSRP	KH	AU-rich element	pri-miR-145, pri-miR-150	miRNA processing

Abbreviations: RBP: RNA-binding protein; RRM: RNA recognition motif; ZF: zinc finger domain; CSD: cold-shock domain; KH: K homology domain.

## Data Availability

Not applicable.
